# Psychosocial problems in children with congenital adrenal hyperplasia in Cipto Mangunkusumo Hospital, Jakarta, Indonesia

**DOI:** 10.1186/1687-9856-2013-S1-P122

**Published:** 2013-10-03

**Authors:** I Nyoman Arie Purwana, Frida Soesanti, Aman B Pulungan, Jose RL Batubara

**Affiliations:** 1 Division of Endocrinology, Department of Pediatrics, Cipto Mangunkusumo Hospital, Jakarta, Indonesia

## 

Congenital Adrenal Hyperplasia (CAH) is a chronic illness that requires lifelong medication and, in some cases, frequent hospitalizations. This situation will bring psychosocial consequences for the patients and their families. This study aims are to determine the psychosocial problems in children with CAH in Cipto Mangunkusumo Hospital.

Data was taken from medical records in Pediatrics Endocrinology Clinic of Cipto Mangunkusumo Hospital, Jakarta, Indonesia during 2007-2012. Out of 96 patients were diagnosed as CAH, 76 patients aged 4-16 years included in this study. Patient’s parents were interviewed by telephone for screening of psychosocial problems using the Pediatrics Symptom Checklist-17 (PSC-17). Patients and their parents were also asked about their expectations in the future.

Out of 76 patients, Twenty five patients had 46,XX karyotype, 2 patients had 46,XY karyotype, while 49 patients have no karyotipe data available. Most children were raised in accordance with the results of their karyotype finding. Few parents reported some behaviour problems with their children according to PSC-17.

Our study suggests that few children with CAH had a psychosocial dysfunction that might be related to their physical condition. Improving knowledge and encouraging parent to join with CAH support group were important to help families with CAH in Indonesia.

